# Intestinal parasitic infections: telephone health literacy with men in areas of poverty in the syndemic

**DOI:** 10.1590/0034-7167-2022-0300

**Published:** 2023-10-09

**Authors:** Julio Cesar Pegado Bordignon, Antonia de Castro Ribeiro, Érica Tex Paulino, Maria de Fatima Leal Alencar, Marcio Neves Boia, Antonio Henrique Almeida de Moraes

**Affiliations:** I Fundação Oswaldo Cruz. Rio de Janeiro, Rio de Janeiro, Brazil; II Universidade do Estado do Rio de Janeiro. Rio de Janeiro, Rio de Janeiro, Brazil

**Keywords:** Intestinal Diseases, Parasitic, Men, Poverty Areas, Health Education, Primary Care Nursing, Parasitosis Intestinales, Hombres, Áreas de Pobreza, Educación en Salud, Enfermería de Atención Primaria, Enteropatias Parasitárias, Homens, Áreas de Pobreza, Educação em Saúde, Enfermagem de Atenção Primária

## Abstract

**Objectives:**

to assess Popular Health Education practices on intestinal parasites, carried out by telephone contact with men living in urban communities in Rio de Janeiro, Brazil, during the COVID-19 syndemic.

**Method:**

a quasi-experimental, quantitative and descriptive study, carried out with men aged 20 to 59 years. Pre-test was applied, and participants were divided into two groups: control and experimental. Popular Education in Health practices were carried out with an experimental group, and post-test was applied for both.

**Results:**

health education practices were significant in the experimental group, with a reduction in incorrect answers. There was an increase in incorrect answers in the control group’s post-test.

**Conclusions:**

the Brazilian National Policy for Popular Education in Health contributed to qualify men’s health literacy on intestinal parasites. Practice by telephone contact proved to be a powerful strategy for nursing and public policies to access this group and promote health in Primary Health Care.

## RESUMO

**Objetivos:**

avaliar práticas de Educação Popular em Saúde sobre parasitoses intestinais, realizadas por contato telefônico com homens residentes em comunidades urbanas do Rio de Janeiro, Brasil, durante a sindemia de COVID-19.

**Métodos:**

estudo quase-experimental, quantitativo e descritivo, realizado com homens de 20 a 59 anos. Foi aplicado o pré-teste, e os participantes, distribuídos em dois grupos: controle e experimental. Foram realizadas práticas de Educação Popular em Saúde com grupo experimental e aplicado o pós-teste para ambos.

**Resultados:**

práticas de educação em saúde foram significativas no grupo experimental, com redução das respostas incorretas. Houve aumento de respostas incorretas no pós-teste do grupo controle.

**Conclusões:**

a Política Nacional de Educação Popular em Saúde contribuiu para qualificar a literacia em saúde de homens sobre parasitoses intestinais. A prática por contato telefônico mostrou-se uma potente estratégia para a enfermagem e as políticas públicas acessarem este grupo e promoverem saúde na Atenção Primária à Saúde.

## RESUMEN

**Objetivos:**

evaluar las prácticas de Educación Popular en Salud sobre parásitos intestinales, realizadas por contacto telefónico con hombres residentes en comunidades urbanas de Río de Janeiro, Brasil, durante la sindemia de la COVID-19.

**Métodos:**

estudio cuasiexperimental, cuantitativo y descriptivo, realizado con hombres de 20 a 59 años. Se aplicó la preprueba y se dividió a los participantes en dos grupos: control y experimental. Se realizaron prácticas de Educación Popular en Salud con un grupo experimental y a ambos se les aplicó la posprueba.

**Resultados:**

las prácticas de educación en salud fueron significativas en el grupo experimental, con reducción de respuestas incorrectas. Hubo un aumento de respuestas incorrectas en la posprueba del grupo control.

**Conclusiones:**

la Política Nacional de Educación Popular en Salud contribuyó a cualificar la alfabetización en salud de los hombres sobre parásitos intestinales. La práctica por contacto telefónico demostró ser una poderosa estrategia de enfermería y de políticas públicas para acceder a ese grupo y promover la salud en la Atención Primaria de Salud.

## INTRODUCTION

The COVID-19 syndemic^(1)^ has led to a high mortality rate and social inequalities, especially in areas with socio-environmental vulnerabilities, where inequalities are exacerbated. These facts contribute to the overlapping of priorities for infectious and neglected disease prevention and, consequently, for poverty maintenance. In this context, intestinal parasitic infections, also known as intestinal parasites, are neglected diseases that mainly affect residents of areas with socio-environmental vulnerabilities in developing countries. They are caused by helminths or protozoa and transmitted mainly by the fecal-oral route, through contaminated water and food, causing, in addition to mortality in more vulnerable areas, morbidities that can contribute to absence from work, to reduce individuals’ productivity and impact the family economy, leading to the loss of 39 million disability-adjusted life years (DALYs) in the world population^(2-3)^.

In Brazil, most intestinal parasites are not notifiable diseases^(4)^, leading to prevalence rates based on dubious estimates or specific studies, and approaches to control these diseases do not prioritize the adult population^(5)^, which contributes to transmission maintenance. In a recent survey^(6)^, it was shown that, in the Brazilian population, the most prevalent parasites are the helminth *Ascaris lumbricoides* and the protozoan *Giardia intestinalis*, with prevalence ranging from 5% to 70% and from 4.9% to 96.6%, respectively. In the state of Rio de Janeiro^(7)^, the overall prevalence ranges from 18.3% to 66%. In *Complexo de Favelas de Manguinhos* (CFM)^(8)^, in the city of Rio de Janeiro, the general prevalence of intestinal parasites was 29.4%, being more frequent in men (31.5%).

Popular Health Education can be a powerful practice^(9)^ to qualify the health literacy of men living in vulnerable areas about intestinal parasites, expanding the ability to make basic decisions to face these diseases, since health literacy is understood as the ability of individuals to obtain, process and interpret health information to make appropriate decisions regarding health care^(10)^. Thus, nurses who work in Primary Health Care (PHC) in the Unified Health System (SUS - *Sistema Único de Saúde*), articulated with a multidisciplinary team and having as a fundamental tool the bond they have with and in the territory, can contribute to the construction of knowledge with the population and qualify health literacy for intestinal parasite prevention or control based on the local reality.

It should be noted that the Brazilian National Policy for Popular Education in Health, instituted by Ministry of Health Ordinance 2761 of November 19, 2013^(11)^, structured in dialogue with popular movements and in Paulo Freire’s epistemological and pedagogical foundations^(12)^, understands Popular Education in Health as “a political-pedagogical practice that permeates actions aimed at the promotion, protection and recovery of health, based on the dialogue between the diversity of knowledge, valuing popular knowledge, ancestry, encouraging production individual and collective knowledge and their insertion in the SUS”, having dialogue, loveliness, problematization, the shared construction of knowledge and the commitment to the construction of a popular democratic project, to be carried out with the assisted population for emancipation in the management of self-care in health, as principles.

On the other hand, the skills and competencies used by an individual in the meaning of health information have three distinct levels of health literacy^(13)^: i) functional literacy, which is related to the way individuals read and assimilate health information, as well as the skills and competencies used to operate numbers, approaching what is understood as health literacy; ii) interactive literacy, which encompasses cognitive skills together with social skills to be actively used in everyday situations, allowing knowledge of communication processes and interrelations that involve the search, interpretation, assessment and meaning of messages about health; and iii) critical literacy, which is related to the set of cognitive and social skills that individuals use to contextualize, qualify and question health information to which they have access, seeking greater control over various issues related to the care of their own health, of others, or of their relationships with different health institutions.

Despite the efforts of some professionals to carry out Popular Health Education practices in PHC’s daily life^(9,14)^, the presence of men is usually discreet, since the habit of self-care is not part of male culture, and unproductivity and disability stigma makes men deny the illness and neglect self-care^(15)^. However, approaches to include men in SUS care practices, through the Brazilian National Policy for Comprehensive Care for Men’s Health, are still insufficient and need to be reviewed, since health education practices are carried out, in mostly vertically, based on male morbidity and mortality data, and that issues associated with social class, race and gender, potentiate health inequities that make men invisible as subjects of law for public policies, not being objects of attention in the context of comprehensive health for the State^(16-17)^.

In addition to the difficulties, obstacles and resistance of the male population to the specificities of “being a man” in coping with the health-disease process, it is worth noting that there are still few studies on care practices for this group^(18)^. It is necessary to consider new strategies that can be carried out by nurses belonging to a multidisciplinary team to face intestinal parasites in the context of PHC. In the current context of a COVID-19 syndemic, these strategies still need to be adapted to the protocols for preventing the transmission of this virus, based on the reality and local culture, on the experiences and knowledge of men living in territories with socio-environmental vulnerabilities, in order to build new knowledge with participants and encourage, through Popular Education in Health, the social role in the control of intestinal parasites, whose prevalence can produce consequences that make community health unfeasible.

## OBJECTIVES

To assess Popular Health Education practices on intestinal parasites, carried out by telephone contact with men living in urban communities in Rio de Janeiro, Brazil, during the COVID-19 syndemic.

## METHODS

### Ethical aspects

This study was approved by the Research Ethics Committee of Oswaldo Cruz Institute (*Instituto Oswaldo Cruz*)/Fiocruz, in compliance with Resolutions 466/2012 and 510/2016 of the Brazilian National Health Council. All participants registered in the research signed the Informed Consent Form.

### Study design, period, and place

We used the SQUIRE instrument to guide the research^(19)^. This is a quasi-experimental, quantitative and descriptive study^(20)^. The study took place from February 2018 to April 2021 at CFM, located in the Metropolitan Region of the city of Rio de Janeiro (22°52’47,04”S - 43°14’57,18”W).

The first phase of the study, which took place from February 2018 to December 2019, focused on patient registration; in the application of pre-test questionnaires; in the analysis of the socioeconomic characteristics of those registered; in CFM’s environmental characteristics; in the coproparasitological inquiry to elaborate Popular Education in Health practices based on the reality experienced by participants; in the frequency of intestinal parasites present among those registered; and risk factors for intestinal parasite transmission in this territory^(21)^. The last phase took place from March 2020 to April 2021, when individual Popular Education in Health practices were carried out by telephone contact for subsequent application of post-test questionnaire and data analysis.

CFM is an area with socio-environmental vulnerabilities, which has low Social (0.473) and Human (0.726) Development Indexes^(22)^. It is subdivided into five large areas with distinct socio-environmental characteristics^(8)^ and, according to the Prime *Saúde* software (version 2.1.87, *Eco-Empresa de Consultoria e Organização em Sistemas e Editoração Ltda*., Rio de Janeiro, RJ, BR), in 2017, it covered approximately 17 thousand households with 42 thousand inhabitants, of which 36.66% were men aged 20 to 59 years.

In a recently published article^(21)^, in the sample of 624 men living in CFM, most participants reported having their own home (66.9%), with ceramic floors (92.5%), toilets with a reservoir (98.2%), supply of water by the public system (96.5%), water storage in a water tank (60.7%) and a filter for human consumption water (66.2%). However, the prevalence of intestinal parasites was 23.8% and about 40% of households had water unfit for human consumption.

### Research subject selection

The StatCalc tool of Epi-Info software (version 7.2.5, Centers for Disease Control and Prevention, Atlanta, GA, USA) was used for sample calculation, considering the number of men aged 20 to 59 years who lived in CFM. According to the study published in 2017^(8)^, a frequency of 30% of infected men was estimated. A confidence level of 95% and a margin of error of 5% were considered. Thus, the study’s estimated “n” was 618 participants, and the proportions of families/households distributed in the five Major Areas were respected in sampling systematics.

Male individuals, living in CFM, aged between 20 and 59 years old and registered for assistance in the Family Health Strategy teams in the territory were included. Residents who had some cognitive disability to answer the survey questionnaires were excluded.

### Study protocol

Pre-test took place from February 2018 to December 2019. Participants were approached by the research team at CFM on the streets, in social facilities in the communities or in their respective homes. In this approach, the research was explained and how the participation would be. Then, the resident was registered by signing the Informed Consent Form and applying the socioeconomic questionnaire, with questions involving respondent data such as age, education and family income^(22)^. Then, the Questionnaire on Knowledge, Attitudes and Practices was applied, with openand closed-ended questions about etiology, forms of transmission, symptomatology, diagnosis and strategies for intestinal parasite prevention^(23-25)^. After the questionnaires were applied, participant coproparasitological survey was carried out to identify the intestinal parasites circulating in CFM and treat these parasites. To elaborate Popular Education in Health practices, the situational diagnosis carried out in the first phase of the study was considered^(21)^.

After this initial phase, participants were divided into two groups, experimental and control, having as a criterion the availability of these individuals to participate in the intervention on the days previously scheduled for Popular Education in Health practices about intestinal parasites. However, due to the social distance necessary to prevent COVID-19 transmission during the syndemic, which has occurred since March 2020^(26)^, such practices were carried out by nurses from the multidisciplinary research team individually, synchronously and remotely by telephone contact^(27)^.

To carry out the strategy chosen in Popular Education in Health practices, participants’ low education and income levels were considered, in addition to the possibility of real-time interaction between them and research team nurses during the activities. Conventional calls were chosen, as some participants could have difficulties accessing or handling free instant messaging applications, which could cause losses for the study. Up to three calls were made per participant from March 2020 to April 2021, on different days and times, to carry out Popular Education in Health practices. Such practices were based on qualified listening and recognition of popular knowledge based on Briceño-León^(28)^, and addressed, in a dialogical, problematizing and reflective manner^(29)^, the concepts related to parasites’ life cycle, diagnosis and strategies for intestinal parasite transmission prevention and control^(30)^.

At the beginning of the contacts, nurses introduced themselves and informed the purpose of the call. The questions *“How does a person develop worms?”, “Where are the worms after they enter people?”, “What do people feel when they have worms?”, “Where do worms go when they come out of people?”, “What do you do to not develop worms?”, What happens to the worm outside the body?”, “How do you identify the presence of worms in people?”* guided the dialogues of Popular Education in Health practices with participants. After each question was asked, the necessary time was given for participants to answer and, upon completion of the answer, or absence thereof, the dialogue explanation was performed on each topic addressed.

During the practices, participants’ autonomy was encouraged to face these diseases in the home and community environments, through the reflection that was carried out regarding the responsibility of the resident regarding the care of their home and peridomicile, aiming at the individual, family and community well-being. The dialogue conducted was contextualized based on the profile of pre-test answers and on everyday situations in CFM communities so that participants could relate the theme to experiences and prior knowledge of this practice^(31)^.

With the experimental group, Popular Education in Health practices were carried out and, at the same time, it was agreed that a new telephone contact would be made via cell phone after 30 days for post-test application. With the control group, post-test application occurred simultaneously with the experimental group activities, but Popular Education in Health practices occurred at the end of post-test application, thus guaranteeing that the control group participants had the same intervention.

### Analysis of results, and statistics

The information obtained through the socioeconomic questionnaire and the Questionnaire on Knowledge, Attitudes and Practices were plotted in a Microsoft Access database (version 2010). In order to carry out the analyzes related to the Questionnaire on Knowledge, Attitudes and Practices, participants’ multiple answers were categorized as (i) correct, (ii) partially correct and (iii) incorrect, according to the answer key adapted from the publication by Ignacio et al.^(25)^ (Chart 1).

**Chart 1:** Answer key for the categorization as “correct”, “partially correct” and “incorrect” of answers of men living in *Complexo de Favelas de Manguinhos*, Rio de Janeiro, Rio de Janeiro, Brazil, to the Questionnaire on Knowledge, Attitudes and Practices about intestinal parasites - February 2018 to December 2019 and March 2020 to April 2021

Questions	Answers		
	Correct	Partially correct	Incorrect
*How does a person develop worms?*	Fecal-oral route and through the skin; may also include zoonotic transmission.	Only one route mentioned or incomplete answers such as not washing hands, walking barefoot and not washing food.	No answer, unknown, or any alternative answer (e.g., a specific food, sexual contact only).
*Where are the worms after they enter people?*	Intestines and other organs related to intestinal parasites’ life cycles (e.g., lungs or liver).	Mentions the intestines.	No answer or unknown.
*What do people feel when they have worms?*	Citing at least two of the following symptoms: diarrhea, abdominal pain, bloating, nausea, anemia, intestinal obstruction, anal itching, asymptomatic.	Only one sign or symptom mentioned.	No answer or unknown.
*Where do worms go when they come out of people?*	Soil, sewage or water.	Mention one of the destinations or just “remains alive”.	No answer, unknown or any alternative answer (e.g., air).
*What do you do to not develop worms?*	Includes forms of prevention that interrupt two transmission routes: fecal-oral, through the skin and/or zoonotic.	Interruption of only one transmission route or incomplete answer.	No answer, unknown or any other method that should not be used as a form of prevention (e.g., taking medication, avoiding sweets).
*What happens to the worm outside the body?*	Mentions that it continues the life cycle in the environment or can infect other people.	It simply mentions “remains alive”.	“Dies”, any alternative answer or no answer.
*How do you identify the presence of worms in people?*	Laboratory (coproparasitological examination or stool sample). It may also include clinical examination.	Only clinical examination; “exam” without specifying the type of exam; or naming signs and/or symptoms.	Any other tests mentioned without including coproparasitological, no answer or unknown.

Descriptive statistics of variables assessed in the study were performed. The analyzes of the changes that occurred were carried out with the same respondents who participated in the two moments of this study. The Wilcoxon test was performed to assess the differences between the two moments (pre and post-test) in relation to the intervention in the experimental group. The Mann-Whitney test was performed to compare the control and experimental groups after the intervention. All analysis was performed in the R software (version 4.1.2, R Core Team - R Foundation for Statistical Computing, Vienna, AUT), with an α significance level of 5%.

## RESULTS

A total of 624 men living at CFM were registered in the pre-test, but 257 participated in the post-test, 144 in the control group and 113 in the experimental group. Of the post-test participants in the control group, 36.8% (53/144) were aged 30 to 39 years, 41.7% (60/144) had incomplete elementary school, and 36.1% (52/144) had an income of two to four minimum wages. Among post-test participants in the experimental group, 34.5% (39/113) were aged 40 to 49 years, 42.5% (48/113) had incomplete elementary school, and 39.8% (48/113) had an income of two to four minimum wages (Table 1). The Mann-Whitney test showed that the control and experimental groups are statistically homogeneous for each assessed aspect (p-value>0.05).

**Table 1:** Associations between socioeconomic variables and the control and experimental groups composed of men living in communities in *Complexo de Favelas de Manguinhos* **, Rio de Janeiro, Rio de Janeiro, Brazil - February 2018 to December 2019 (pre-test) (N= 257)**

Aspects assessed	Control	Experimental	Total	*p* value
	n (%)	n (%)	n (%)	
Age				0.887
20-29	20 (44.4)	25 (55.6)	45 (100)	
30-39	53 (71.6)	21 (28.4)	74 (100)	
40-49	33 (45.8)	39 (54.2)	72 (100)	
50-59	38 (57.6)	28 (42.4)	66 (100)	
Education				0.474
Illiterate	5 (71.4)	2 (28.6)	7 (100)	
Incomplete elementary school	60 (55.6)	48 (44.4)	108 (100)	
Complete elementary school	19 (63.3)	11 (36.7)	30 (100)	
Incomplete high school	15 (65.2)	8 (34.8)	23 (100)	
Complete high school	38 (49.4)	39 (50.6)	77 (100)	
Incomplete higher education	4 (50.0)	4 (50.0)	8 (100)	
Complete higher education	3 (75.0)	1 (25.0)	4 (100)	
Family monthly income				0.230
Less than 1 minimum wage	7 (70.0)	3 (30.0)	10 (100)	
1 minimum wage	39 (54.2)	33 (45.8)	72 (100)	
1-2 minimum wages	32 (68.1)	15 (31.9)	47 (100)	
2-4 minimum wages	52 (53.6)	45 (46.4)	97 (100)	
More than 4 minimum wages	10 (43.5)	13 (56.5)	23 (100)	
Did not answer	1 (33.3)	2 (66.7)	3 (100)	
Does not know	3 (60.0)	2 (40.0)	5 (100)	
Total	144 (56.0)	113 (44.0)	257(100)	

p value referring to the Mann-Whitney test; minimum wage: BRL 998.00.

The most frequent moments in which participants preferred to schedule contacts were during their lunch break during office hours, after working hours, or on weekends. In Popular Health Education practices, most participants interacted during the dialogue about preventive measures for intestinal parasites, with questions regarding care with food preparation, home infrastructure and personal habits, mainly related to hand hygiene. The practices lasted, on average, 20 minutes and some participants took advantage of the moment to report any health complaints or resolve any doubts regarding access to services offered in PHC.

**Figure 1:**
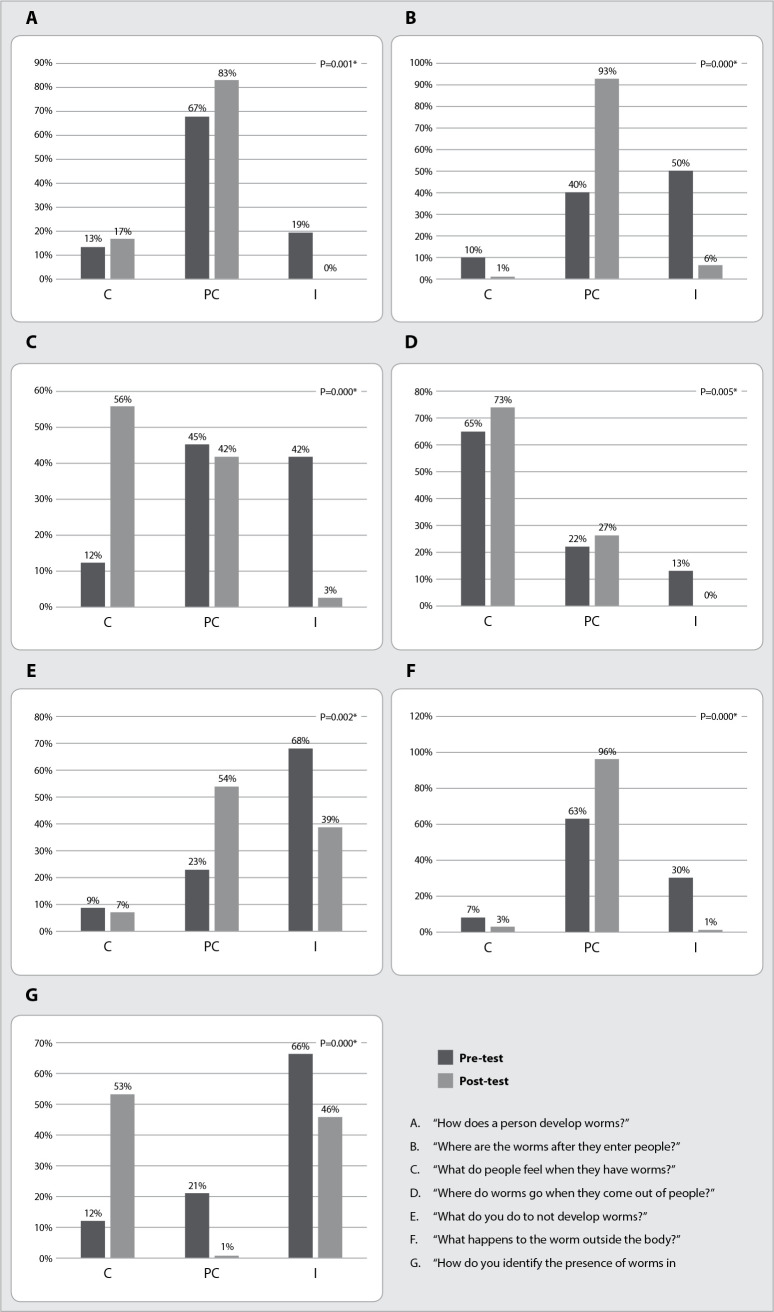
Comparison between answers of men living in *Complexo de Favelas de Manguinhos*, Rio de Janeiro, Rio de Janeiro, Brazil, to the Questionnaire on Knowledge, Attitudes and Practices about intestinal parasites - February 2018 to December 2019 and March 2020 to April 2021. Experimental group (pre and post-test) (n=113)

In the experimental group, it was possible to identify that all the answers were statistically significant as a result of the intervention of Popular Education in Health practice in a synchronously and remotely, carried out by telephone contact, reducing the number of incorrect answers (Figure 1).

p value referring to the Wilcoxon test; *for significant values; C - correct answers; PC - partially correct answers; I - incorrect answers.

**Figure 2:**
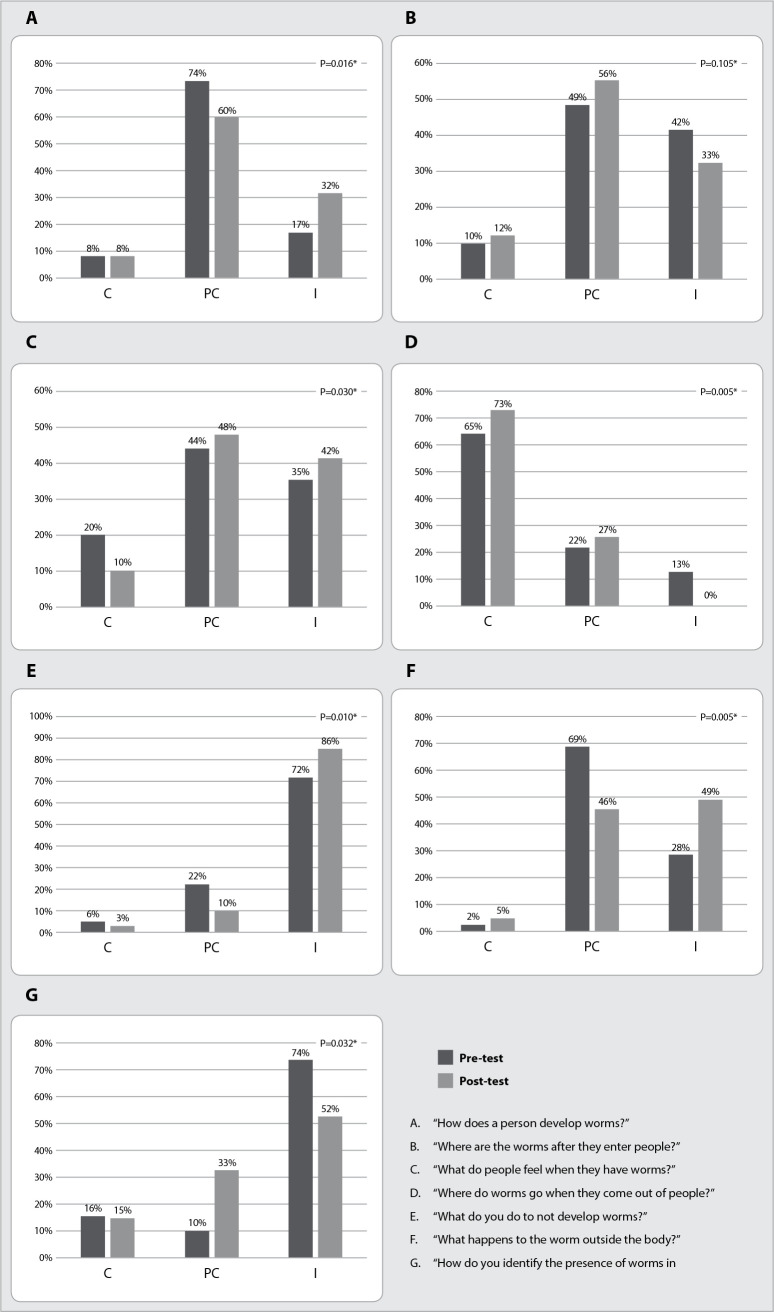
Comparison between answers of men living in *Complexo de Favelas de Manguinhos*, Rio de Janeiro, Rio de Janeiro, Brazil, to the Questionnaire on Knowledge, Attitudes and Practices about intestinal parasites - February 2018 to December 2019 and March 2020 to April 2021. Control group (pre and post-test) (n=144)

In the comparison between preand post-tests of control group participants, it was possible to observe variations in answer classifications to the Questionnaire on Knowledge, Attitudes and Practices about intestinal parasites. Significant differences were observed in the answers between the preand post-test period for six of the seven items assessed in the Questionnaire on Knowledge, Attitudes and Practices. In post-test, an increase in incorrect answers was observed (Figure 2).

p value referring to the Wilcoxon test; *for significant values; C - correct answers; PC - partially correct answers; I - incorrect answers.

**Figure 3:**
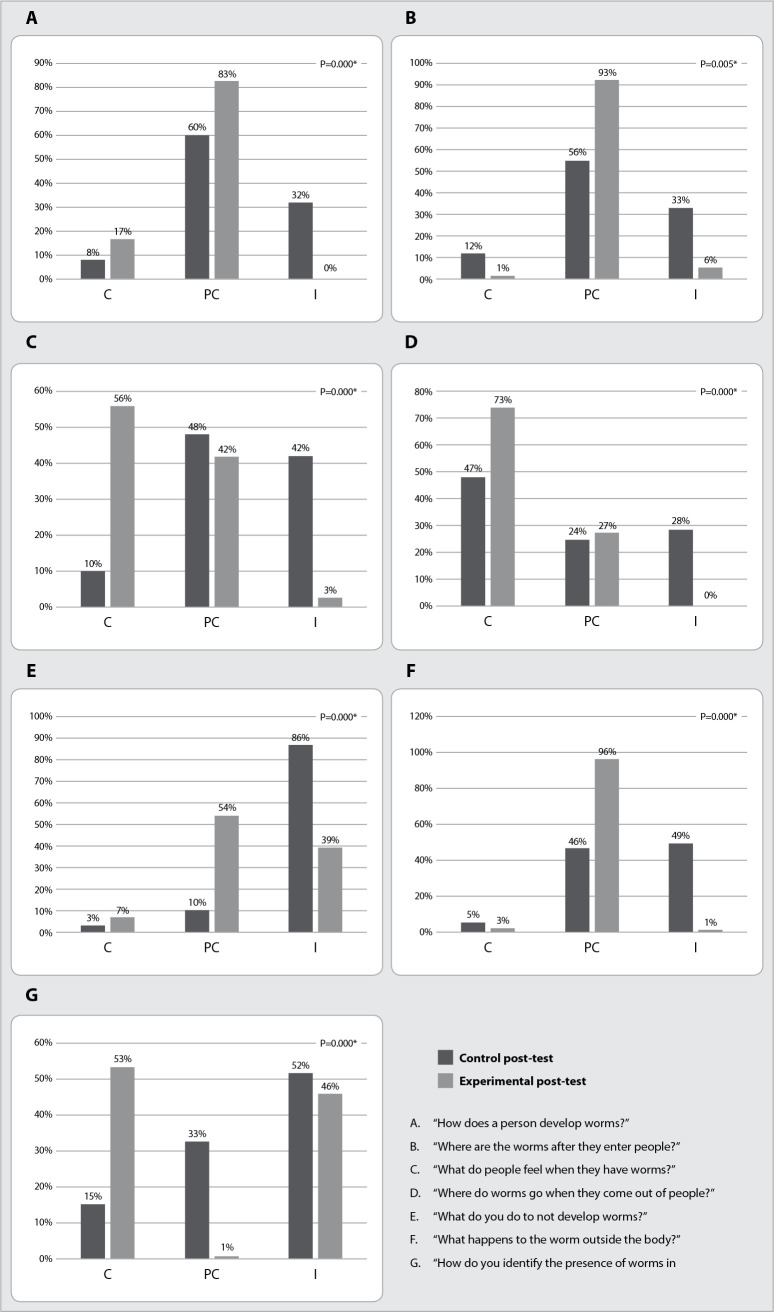
Comparison between answers of men living in communities in *Complexo de Favelas de Manguinhos*, Rio de Janeiro, RJ, Brazil, to the Questionnaire on Knowledge, Attitudes and Practices about intestinal parasites - March 2020 to April 2021. Post-test, control (n=144) and experimental (n=113) groups

The answers of control experimental group participants were compared to the post-test. Even though the control group had not experienced the intervention of Popular Education in Health prior to the second application of the Questionnaire on Knowledge, Attitudes and Practices about intestinal parasites, the two groups showed significant differences (p<0.00) in the answers of all the assessed questions. Experimental group participants had fewer incorrect answers than the control group (Figure 3).

p value referring to the Mann-Whitney test; *for significant values; C = correct answers; PC = partially correct answers; I = incorrect answers.

## DISCUSSION

The discreet participation of men living in urban communities in face-to-face Popular Education in Health practices was what motivated this study, since the strategies carried out in PHC that contemplate them for care with the SUS are minimal. According to Separavich & Canesqui^(17)^, men limit health care to physical exercise practices, observance of diet and seeking assistance in extreme situations. These notes support the need to update public policies for greater inclusion of men in SUS care practices, as well as the institutionalization and recognition of the Brazilian National Policy for Popular Education in Health for greater effectiveness of educational practices, in view of its principles, strategic axes and specific objectives^(11-12)^.

The reduced presence of men in health education practices was also observed by Mourão et al.^(32)^. Participating men report that they do not feel included in health promotion actions, as they are carried out in a general way, including men and women, and aimed at a certain health issue. The lack of management support and an appropriate infrastructure were signaled by health professionals. This suggests that, even with the Brazilian National Policy for Popular Education in Health, in force since 2013^(11)^, there is still little health professionals’ and managers’ encouragement and/or preparation to put it into practice, which contributes to poor health literacy, which is often accompanied by shame and less ability to understand how to prevent disease and promote health^(13)^.

For Souza and Souza et al.^(33)^, men are created not to show feelings and weaknesses towards society. The search for health care and/or the discovery of a disease goes against this culture, with men having their masculinity contested. Linked to this, the barriers in the search for the health service, given by the incompatibility between the hours available for men and the Basic Health Units’ operation hours, due to delay in care and lack of educational practices and/or specific care for men’s demands, they contribute to their seeking care at emergency services that are open 24 hours a day. Similarly, health professionals’ work, with an imposing posture and hospital-centered attitudes, also becomes an obstacle to the presence of men in PHC services^(33)^, pointing to the need for professional training and adaptations in the service to welcome and meet men’s health needs.

Of the 624 men living in CFM registered for this study, it was possible to capture a total of 257 participants synchronously and remotely by telephone contact. This fact can be justified mainly due to the high impact of the COVID-19 disease on the CFM population, given the worsening of poverty, inequalities and pre-existing social vulnerabilities upon arrival and with the advance of the COVID-19 syndemic^(1)^, when many participants may not have been able to keep their respective telephone lines active, which prevented access to them. However, changing the phone number, the phone being turned off, not answering calls or giving up participating in the research may also have favored the loss of participants^(34)^.

Based on the answers to the socioeconomic questionnaire, it was possible to observe that among participants, low education predominated, which may contribute to the reduced demand for PHC services. Men’s low educational level, when linked to the barriers to access and accessibility to the services of Basic Health Units, such as incompatibility between the opening hours of these units and the working hours of most Brazilian men, strenuous working hours and working conditions return home, can enhance insufficient health literacy^(13)^. These data corroborate those of Silva & Melo^(16)^ and Separavich & Canesqui^(17)^, since men are not in the habit of looking for preventive care and tend to seek assistance in services where demands are answered objectively.

It was also observed a lack of basic knowledge of participants in pre-test of both groups regarding preventive measures for intestinal parasites, which may be associated with the low level of education reported and the high prevalence of these diseases in the territory, since they may not know the measures to interrupt the transmission cycles of these diseases or do not associate them with daily practices^(8)^, due to the likely functional level of health literacy they have^(13)^.

Regarding the association made between the socioeconomic variables and the control and experimental groups, no statistical significance was observed with any of these groups, showing the homogeneity between them. According to Said & Salem^(35)^, the absence of statistical significance between the answers on the subject before the intervention in education is carried out is one of the criteria that can show the effectiveness of health education practices by telephone contact. Socioeconomic characteristic homogeneity ensures that there was no bias in participant selection in each study group and that significant differences between answers before and after the intervention in the experimental group show that Popular Education in Health practice by telephone contact was able to have positive effects in the studied population during the COVID-19 syndemic, which still affects and economically impacts the CFM population. These effects may be more noticeable and expressive in recurrent and face-to-face practices involving PHC professionals and the CFM population, especially when social distancing is no longer considered a protective measure for COVID-19 transmission.

When comparing the preand post-tests of individuals in the control group, it was possible to observe that there was an increase in answers classified as “incorrect” in the second moment. This fact suggests that, over time, the absence of dialogue about intestinal parasites between health professionals and the population can contribute to the increase in knowledge gap and insufficient health literacy about these diseases, allowing transmission maintenance and the high prevalence existing in CFM. Bragagnollo et al.^(31)^ obtained similar results in a group of students from São Paulo and emphasized the need for action by the three spheres of government to control these diseases.

With regard to Popular Education in Health practice synchronously and remotely with the experimental group, it was possible to observe an improvement in the quality of participants’ answers in post-test, given the significant reduction in answers classified as “incorrect”. These data corroborate the results of Pereira et al.^(36)^ who, when assessing the effects of the intervention on the knowledge of residents of other communities in the state of Rio de Janeiro, also observed a reduction in “incorrect” answers regarding symptomatology, parasites’ life cycle and prevention measures.

When comparing the post-test answers of the control group with the answers of the experimental group, it was possible to observe significant differences (p<0.00) in all questions, which proves the effectiveness of Popular Education in Health practice synchronously and remotely, by telephone contact, for qualification of knowledge and health literacy of men living in CFM about intestinal parasites. Telephone contact use was also effective in educational interventions regarding support for women during the breastfeeding period^(34,37)^.

According to Shao et al.^(38)^, technology use during the COVID-19 syndemic was of great importance, as they facilitated access to information and communication over long distances. In this regard, telephone contact proved to be a valuable tool to be used in the SUS, as it is low cost and does not require an internet network, being a powerful access strategy for men to strengthen the bond with the unit. Basic Health Care through nurses or other PHC professionals.

### Study limitations

The necessary social distancing, the impacts on CFM population’s health and the economic crisis resulting from the COVID-19 syndemic were limitations that must be considered when interpreting the results. As for registered men, 58.8% (367/624) did not participate in post-test activities, with 53.8% (168/312) belonging to the control group and 63.8% (199/312) to the experimental group.

Since it is a quasi-experimental and quantitative study, there are implications in the potential for generalization of results, which may be less conclusive or there may be alternative explanations for the observed results.

The fact that only one Popular Education in Health practice was carried out with each participant by telephone contact should also be considered as a limitation in the interpretation of results.

Although the intervention carried out points to a change in the knowledge of men living in CFM about intestinal parasites, it was not verified whether such interventions were sufficient to generate behavioral changes in the face of these diseases, nor was a new post-test applied to the members of the experimental group to assess whether knowledge was maintained over time.

### Contributions to nursing, health, or public policies

This study on Popular Education in Health practices about intestinal parasites carried out with men living in urban slums synchronously and remotely by telephone contact brought contributions to the context of men’s health, to health professionals’ attitudes and practices and to coping with these diseases within the scope of PHC. These practices allowed men to be closer to services at this level of care and favored dialogue with health professionals, in this case, nurses.

The results of this study can contribute to an update of the Brazilian National Policy for Comprehensive Care for Men’s Health in the face of intestinal parasites, considering the issues that make men invisible to public health policies, the social determination of health, knowledge and popular culture as well as local reality.

## CONCLUSIONS

The Brazilian National Policy for Popular Education in Health contributed to enhance the construction of knowledge about intestinal parasites with men living in urban slums in Rio de Janeiro, through practices carried out by nurses synchronously and remotely, by telephone contact during the COVID-19 syndemic. Thus, telephone contact can be a strategy for nursing and for public policies to access men, and may be considered in a future update of the Brazilian National Policy for Comprehensive Care for Men’s Health as a tool for promoting the health of this group at PHC.

At the same time, Popular Health Education practices by telephone contact proved to be a possible and powerful tool to qualify participants’ health literacy regarding intestinal parasites. The problematization of the lived reality and the shared construction of knowledge for coping with these diseases with men could contribute to the development of skills and competencies capable of positively interfering with self-care management, in knowledge about CFM’s social determinants and in the concepts that involve the social determination of the health-disease process for the studied group.

This method for Popular Education in Health managed, in a simple and effective way, to transpose some paradigms of masculinity established in society regarding health care, since it was elaborated considering these individuals’ availability to participate in the activities, the previous knowledge and the local culture about intestinal parasites. It should be noted that Popular Education in Health by telephone contact can be applied to various health issues and different socioeconomic groups, because it can be adapted to better build knowledge together with the assisted population and thus promote citizens’ autonomy regarding their status as subjects of rights, responsible for their health projects and ways of living life.

## Data Availability

https://doi.org/10.48331/scielodata.JZGUPP
